# Females of the red damselfly *Mnesarete pudica* are attracted to more ornamented males and attract rival males

**DOI:** 10.1038/s41598-020-71057-z

**Published:** 2020-08-31

**Authors:** Paloma Pena-Firme, Rhainer Guillermo-Ferreira

**Affiliations:** LESTES Lab, Hydrobiology Department, Federal University of São Carlos, São Paulo, Brazil

**Keywords:** Behavioural ecology, Animal behaviour

## Abstract

Male calopterygid damselflies often exhibit colourful wings used during aggressive contests and courtship displays. Evidence suggests that male wing coloration is a secondary sexual character assessed by males and females to identify male quality. In some species, males adopt a lekking strategy, where females visit exhibition arenas and choose the best mate. Here, we addressed whether the behaviour of *Mnesarete pudica* males is influenced by female visitation when gathering in leks. We hypothesized that female visitation would increase male investment in courtship and fighting, while reducing patrolling flights and harassment attempts. Moreover, we tested the hypothesis that more ornamented males attract more females to the territory, following the hotshot model of lek evolution. Our results suggest that, indeed, males with more pigmented wings attract more visiting females, independently of male size. Our results also show that the number of females in a territory attracts more males and elicits male contest behaviour, reducing male harassment. We conclude that male ornament and male clustering is a good predictor of female visitation rates, suggesting that females may exert mate choice.

## Introduction

Lek polygyny is a rare mating system across animal taxa, which are defined as display arenas that are defended by males and visited by females for the only purpose of mating^1^. For a species with lekking behaviour to be considered as such, it must exhibit these features: (1) there is no male parental care and males only provide gametes to females; (2) males aggregate in exhibition arenas; (3) males do not defend usable resources to females; (4) males defend territories within the arena where they display to females; and (5) females are able to roam freely through the arena and select a mate^2–4^. Although lek mating systems are rare and usually associated with birds and mammals, several studies have shown that insect species that exhibit lekking behaviour may be more common than has been recognized^5–7^. For instance, some moths^8,9^, fruit flies^5,7^, paper wasps^10^, orchid bees^11^, butterflies^12,13^, and tarantula hawks^14^ are known to exhibit lekking behaviour.

Theoretical models have attempted to explain the evolution of such gregarious behaviour. The ‘hotspot’ model predicts that males will cluster in areas that are constantly visited by females, while the ‘hotshot’ model predicts that males will gather around a high-quality territorial male^15,16^. A third model, the ‘black hole’ model, proposes that females are not attached to specific sites nor to high quality males, but female mobility is random and reacts to male harassment levels^17^. The fourth model, namely the ‘female preference model’, proposed that females prefer to visit larger leks (with more aggregated males)^15^. In the first model (hotspot), female visitation rates should be constant across leks^16^. In any other case, evidence suggests that female visitation rates vary according to lek composition. High female visitation rates may attract more males and increase male-male competition^1^, eliciting male behaviour by increasing male courtship behaviour frequencies^18^ and decreasing harassment levels by males^19,20^.

The lekking behaviour usually consists of male extravagant courtship displays such as behavioural displays, pheromone emission and acoustic signals that attract visiting females^16,21^. Male calopterygid damselflies are famous for their remarkable wing coloration and their fighting and mating rituals^22^. One might find these stream dwellers engaged in long aerial displays against rival males, flying in circles or in ascending face-to-face disputes^23^. Males also exhibit complex courtship displays, showing off their colourful wings to females^24–26^. These highly conspicuous wing colours (for conspecifics and predators, e.g.,^27^) are deemed to be exaggerated male ornaments, which are related with fitness traits^28^. Although males of most species defend territories with useful resources for females to oviposit^26,29–31^, males of some species defend areas that contain no usable resources for females^32^.

Cordoba-Aguilar et al.^32^ suggested that calopterygid damselflies of the genus *Hetaerina* exhibit a lek mating system, since the females do not oviposit inside the male territory. This study considers that lekking behaviour in *Hetaerina* closely resembles the leks in swarming insects, because this genus presents no pre-copulatory female choice since there is no courtship behaviour. The authors also suggest that lekking behaviour in Odonata may be considered primitive, given the ancestral position of Hetaerininae^33,34^. Unlike *Hetaerina*, courtship displays, female choice and resource defence are common among the Calopterygidae genera, suggesting a more derivate state^26^.

Males of the red-winged damselfly *M. pudica* Hagen in Selys (Zygoptera, Calopterygidae) not only engage in complex aerial fights with other males to defend their territory^35^, but also exhibit striking courtship displays to females—the only case in Hetaerininae^24^. Males defend territories with no usable resources for females but fight for perches that can even be located away from the water body where females oviposit alone. Here we addressed whether male behaviour in *M. pudica* follows the ‘hotshot’ model for lekking behaviour, answering whether male sexual ornament predicts female visitation rates. If indeed males follow the ‘hotshot’ model, more females would visit higher quality males (i.e., with more ornamented wings). If this pattern is not observed and female visitation is not predicted by male quality, one might expect that these damselflies follow the ‘hotspot’, ‘female preference’ or ‘black hole’ models. Furthermore, we addressed whether female visitation rates influence male behaviour. We expected that visiting females would:

(i) attract more males to a territory—according to the ‘hotshot’ model, males that are more visited by females should attract more lower quality rivals to the territory;
(ii) increase the number of male-male contests—due to increased number of rivals invading the territory;
(iii) increase male courtship investment—it is known that display frequencies recruit more females, resulting in a positive feedback^36^;
(iv) decrease male harassment (i.e., unsuccessful chasing and mating attempts);
(v) decrease patrolling flights—with increasing male density, male competition should increase, reducing the time budget available for patrolling flights.

## Results

**Figure 1:**
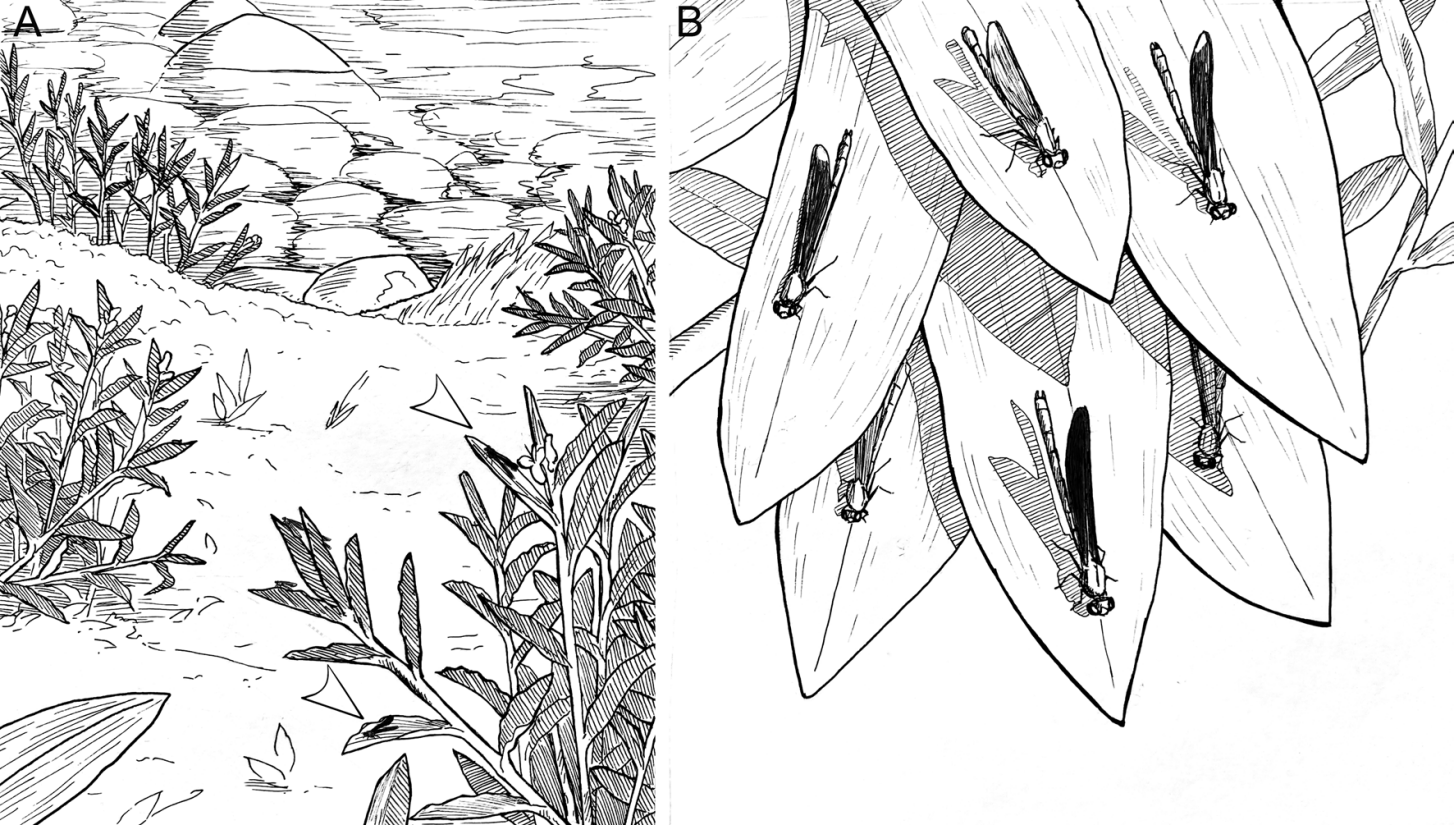
The lekking arena where males of *Mnesarete pudica* aggregate and dance to females during extravagant courtship displays (**A**). Inside the arena, a high-quality male defend a perch (male with darker wing in the centre), where females (clear winged) and other males (two males with dark wings in the upper leaves) aggregate around the territorial male (**B**). Arrowheads point to territorial males perched, separated by 1–2 m. Drawings by Anderson Lepeco.

Males aggregated in a lek arena of ca. 50 m^2^ (a stream patch of 5 × 10 m, Fig. 1a), where focal males defended territories on the vegetation (Fig. 1b). The following results are presented as mean ± SD. Territories were visited by 1.45 ± 1.24 females (range 0–7) and attracted 0.63 ± 0.62 rival males (range 0–3). Focal males harassed females 1.81 ± 2.58 times (range 0–15). Males exhibited 2.45 ± 2.77 patrol flights (range 0–12), 3.35 ± 4.81 contests (range 0–25), and 1.1 ± 1.69 courtship displays (range 0–8).

**Figure 2:**
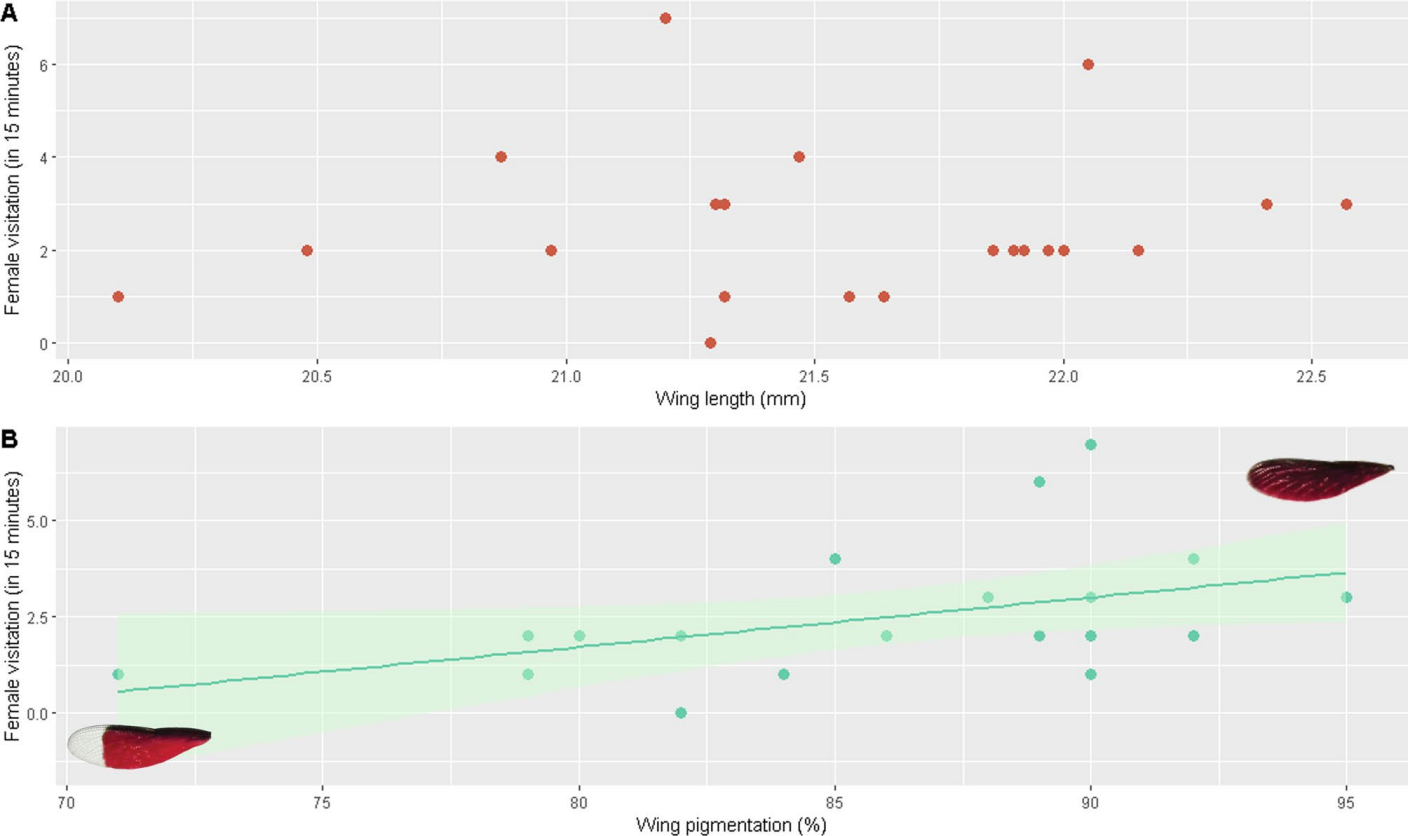
The relationship between male wing length (**A**) and wing pigmentation (**B**) and female visitation (in a period of 15 min) in the damselfly *Mnesarete pudica.* Plotted with *ggplot2* in R.

Male body size (wing length) had no effect on female visitation (GLM Poisson, *β* = 0.36, R^2^ = − 0.054; z = 0.143; p = 0.886, N = 21; Fig. 2a). More females visited males with more pigmented wings (GLM Poisson, *β* = 0.59, R^2^ = 0.131; z = 2.033; p = 0.042, N = 21; Fig. 2b). These results suggest that leks in this species follow the ‘hotshot’ model. There was no relationship between wing length and wing pigmentation (Lineal Model, R^2^ = 0.023, p = 0.238).

**Figure 3:**
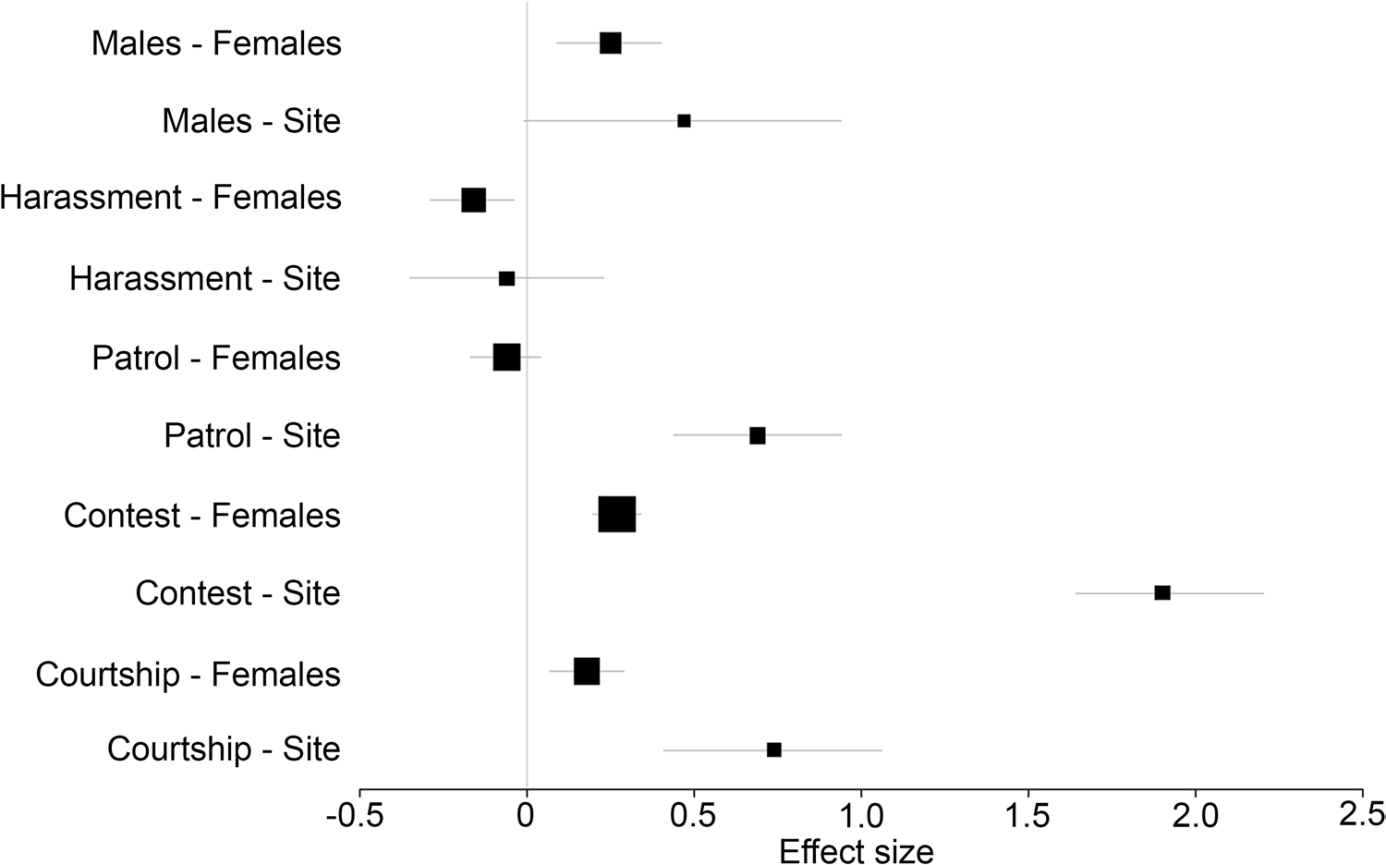
Results of the Bayesian Multivariate GLM with Poisson distribution, showing the mean effect sizes and 95% confidence intervals representing the relationship between male behaviour, female visitation (Females) and site (Site) in lekking arenas of the damselfly *Mnesarete pudica*. Deviations from zero show a significant relationship. Males: the number of males that gathered around the focal male; Females: the number of females that visited the focal male; Site: the site where data was collected (Uberlândia or Assis); Patrol: the number of patrol flights exhibited by the focal male; Contest: the number of contests the focal male was involved; Courtship: the number of courtship displays exhibited by the focal male. Plotted with *brms* and *ggplot2* in R.

Results of the male response according to female visitation and site (Bayesian Multivariate GLM) are presented in Fig. 3, where significant results deviate from zero. Female visitation had a clear impact on male behaviour. Territories with more visiting females attracted more satellite males and increased the number of contests the focal male was involved. As more females visited the territory, males decreased harassment on females and increased courtship displays. Patrolling behaviour was not affected by female visitation. The site of data sampling had significant effects on most behaviours, except for the number of males in the territory and harassment behaviours. These results suggest that the higher female visitation rates attract more males that gather around the focal male, which also decreases harassment intensity, independently of the study site. Patrolling, contesting, and courtship behaviours had higher effect sizes regarding the study site, when compared to the effect sizes of female visitation. These results may indicate that such behaviours are more prone to local environmental variables and population structure than female visitation rates.

## Discussion

Our results show that females of *M. pudica* prefer to visit males with more pigmented wings. It is noteworthy to highlight here that females do not visit a territory because of resources, because *M. pudica* male defend no resource available for females to oviposit, males release the female right after copulation and females oviposit alone elsewhere. Moreover, satellite and territorial males arrive earlier and dispute the best territories before females arrive. Wing pigmentation in calopterygids usually indicates male quality^28,37^ and is evaluated by the females during mate choice^38^, suggesting that there is also pre-copulatory female choice in *M. pudica*^24^. Evidence suggests male *Hetaerina* damselflies do not court females, and that the leks in Odonata would resemble the leks of swarming insects^32^. However, since the males of *M. pudica* exhibit courtship behaviour, like more derived calopterygids such as *Calopteryx*^39^, their behaviour do not match any previously described behaviour for odonates. Lekking behaviour in *M. pudica* may resemble more that of hill-topping insects^6^, rather than swarming insects (which exhibit no courtship behaviour). In some insect species, hill-tops function as rendezvous and lekking arenas^2^. These sites are constantly visited by females and have no resources for females, only males waiting to mate while perched on tall vegetation^2^. Males of *M. pudica* also perch on the top of trees and bushes to display their wings to females^24^, on sites that have no usable resources for females. By following the studied populations for several years, one of the authors (RGF) observed that males always aggregate at the same patch of the stream, like hill-topping insects do^2^.

Our results also show that female visitation and the number of males in a territory have a close relationship. This result may mean that females prefer to visit territories of high quality males, resulting in a cluster of males and females in these territories as occurs in other taxa^6^. In some calopterygids, males may harass females while trying to force copulation when in high densities^40,41^ what can be energetically costly to females^42^. Thus, here we hypothesized that female clustering may reduce the harassment levels^20^. Our results corroborate this hypothesis since males decreased harassment with increased female visitation. Harassment is known to reduce female longevity and reproductive success^42,43^ and to change female investment on egg numbers and size^41^. Moreover, harassment varies seasonally because it is highly dependent on densities and operational sex ratio^44–46^. Hence, females may adopt strategies to reduce harassment, for instance, clustering around males that will repel other males. This would not only reduce harassment by other males, but also reduce harassment by the territorial male that should invest more in courting and defending females in his territory.

We also hypothesized that there should be a positive relationship between the number of females in a territory and male courtship intensity^18,47^, usually because high display frequencies recruit more females^36^. Results show that female visitation increased the number of courtship displays. Nevertheless, courtship was mostly influenced by the study site, as most male behaviours were. It is known that increased male-male competition in one site may make males have a shorter time budget to invest on courtship, hence, some territorial males may change strategy and attempt to force copulations when in high male densities^40^. Therefore, courtship frequencies may be under a higher influence of environmental factors and operational sex ration, than female visitation rates. Our results show that females may reduce harassment by clustering around a chosen male, which may be a strategy to avoid forced copulations.

The effects of female visitation on male clustering may also impact male territorial behaviour. Here, our results suggest that females elicit and increase male contesting behaviour^48^, considering that males tend to invest more in energetic costly agonistic displays when there is a potential return for it^49^. Moreover, the higher number of rivals may increase dispute for females that visit the territory. However, the study site had a higher effect on contest behaviour, suggesting that population or environmental characteristics may be more determinant of male aggressive behaviour. Female visitation had no effect on patrolling flights, but the study site influenced patrolling behaviour, hence, the time the territorial male spends in the territory may respond to environmental conditions, instead of female visitation.

In conclusion, we suggest that the costly ornaments of calopterygid damselflies^28,50^ may function as visual signals to attract females to territories.. Considering that around a high-quality male there will more males and females, corroborating the ‘hotshot’ model, this context increases male-male contests and decreases male harassment on females. Future studies should address the costs and benefits of attracting more females—and consequently rivals—for high quality males. Such studies may provide insightful evidence of a trade-off between male sexual ornaments and female visitation rates, which may increase costs due to increased male-male interactions.

## Methods

### Study species

Males of *M. pudica* arrive early in the morning (between 9 and 10:00 h) and perch on the vegetation in a specific patch of the stream borders, which we called the lekking arena. In this study, males usually perched on leaves of bushes, trees and invasive water lilies (*Hedychium coronarium*) at a height 1.50–2.0 m. Notably, males start fighting for these perches and begin to establish territories and form a kind of exhibition arena that ranges from the stream borders to up to 5 m away from the water.

Lekking arenas are defined as the perches used by males inside a specific area (e.g., 30 cm) or the entire plant used by males^5^. Here, we interpreted the lekking arena as the whole cluster of males on the vicinity of the stream patch, mainly water lilies where males gathered. We considered a territory an area of 1–2 m in diameter^51^ around a territorial male that defend its perch against rivals and constantly patrolled it searching for females and intruders. This distance is presumably a common rule for odonates, where apparently males are able to detect conspecifics^51^. Here, we defined male behaviour as lek because: (i) males do not provide parental care (as all odonates); (ii) males aggregate in mating arenas; (iii) males do not defend oviposition resources—females oviposit alone outside of male territories and males may defend sites 5 m away from the water, with no oviposition resource or access to it; (iv) females are free to visit several male territories in the arena. For terms of consistency, here we used the concept of insect leks presented by Bradbury^4^, Alcock^2,14^ and Shelly^5^ for insects. As proposed by Shelly^5^, we define territory here as an area inside the mating arenas that males hold and defend against rival males where there are no defendable resources (a “symbolic-territory”)^13^. Unfortunately, we were not able to note mating behaviour as we only observed two copulations throughout the study period, which occurred inside the lek.

### Fieldwork

Fieldwork was carried out at one stream in the Ecological Reserve of the “Clube de Caça e Pesca Itororó de Uberlândia” (CCPIU), Uberlândia, Minas Gerais state, Brazil (15° 57′ S, 48°12′ W; altitude 863 m; 640 ha) in March and July 2010, and at another stream in a farm located in Assis, São Paulo state, Brazil (22° 38′ S, 50°27′ W; altitude 522 m) in July 2010. We made behavioural observations from 10:00 (when males begin to fight and court females) to 15:00 (when sexual and fighting activity declines). Males were marked with a unique combination of colours (using nontoxic correction fluid, Faber Castell) on the thorax and abdomen to identify focal males and to prevent sampling the same male multiple times. We used only mid-age males in this study, since young males have black wings and are not sexually matured^52^ and older males lack territorial behaviour^30^. Age estimation was done by checking wing stiffness, since older males have rigid yellowish wings^30^. Wing colour was assessed by eye. Females were marked with a unique number on the wings using an indelible ink pen (Faber Castell).

We made behavioural observations consisting of 15 h of data gathering at each site, using the focal animal method described by Altmann^53^, observing each focal male for 15 min (N = 120 males, 60 males in each site). All males were marked at least 30 min before observations. Observations started when the focal male took flight in a patrol or interacted with another male or a female. This was done because males can spend several minutes in inactivity before a patrol flight. We observed males that were either alone or with another male or a female in the territory. We assume that most of them were marked, and rarely we found a male or female unmarked. When it occurred, we ignored the individual during the 15 min period of focal observations on a marked male, and then collected the unmarked individual. This avoided disrupting the behaviour of the focal male.

During the observations, we noted the focal male behaviour: (a) the number of intraspecific contests; (b) the number of courtship displays; (c) the number of female harassment events; and (d) the number of patrolling flights. Moreover, we noted: (e) the number of visiting females; and (f) the number of rival males in the territory. Courtship displays occurred when a male hovered around a female, perched and “danced” spreading the red wings to the female on the top of a tree or bush^24^. Male harassment occurred when a male dashed towards a female and tried to grab her wings by force and attempted to clasp her with the abdominal appendages. The female usually wards-off the male by spreading her wings and raising abdomen, a refusal or threat display in odonates^54^. Hence, we noted male harassment when a male attempted to force copulation and was refused by the female. A contest occurred when the focal male engaged in an aerial threat display against a rival male, hovering face-to-face or side-by-side in descending and ascending flights^35^. Behaviours were not controlled by male or female visitation, since we were interested in the time budget spent by the focal male on different behaviours in a 15 min time window.

To address whether males with more pigmented wings attract more females, we observed 21 males with the method described above in the Assis population. These males were considered high-quality males, which actively interacted with females and other males. After observations, males were captured and taken to the lab. Wings were dissected and photographed with a digital camera (Canon EOS 70D) along with a scale. We measured the right forewing length and area, and the pigmented area in Adobe Photoshop CS3 to calculate the relative proportion of the wing that is pigmented. This variable (pigmentation area/wing area) was used as an indicator of male quality^35^. We used the forewing because sometimes the hindwing is fully pigmented, while the forewing shows more variation. To know how repeatable these measurements were, two persons measured pigmentation in all males. Both measurements showed a close relationship (r Pearson = 0.918; p < 0.001). We used wing length as a proxy for body size, since it is a reliable measure in damselflies^55^.

### Statistical analyses

To answer whether more pigmented males attracted more females, we built a Generalized Linear Model (GLM) with Poisson loglinear distribution, considering the number of females the dependent variable and male wing pigmentation and wing length as covariate predictors. To answer whether female visitation rates influence the focal male behaviour, we built a Bayesian Multivariate GLM with Poisson loglinear distribution, where the number of females and site of collection were considered covariate predictors and the number of courtship displays, harassment attempts, male-male contests and patrolling flights were considered dependent variables. Analyses were performed using the packages *brms*^56^, and figures were plotted using *ggplot2*^57^, both in R environment^58^.

## Data Availability

The datasets generated during and/or analysed during the current study will be available in the Dryad depository upon publication.
